# High-dose thiamine supplementation ameliorates obesity induced by a high-fat and high-fructose diet in mice by reshaping gut microbiota

**DOI:** 10.3389/fnut.2025.1532581

**Published:** 2025-02-07

**Authors:** Yu Xia, Lulu Wang, Yanyan Qiu, Weihong Ge

**Affiliations:** ^1^Department of Pharmacy, China Pharmaceutical University Nanjing Drum Tower Hospital, Nanjing, China; ^2^School of Basic Medicine and Clinical Pharmacy, China Pharmaceutical University, Nanjing, China; ^3^Department of Pharmacy, Nanjing Drum Tower Hospital, The Affiliated Hospital of Nanjing University Medical School, Nanjing, China; ^4^Department of Pediatrics, The First Affiliated Hospital of Guangxi University of Chinese Medicine, Nanning, China

**Keywords:** thiamine, obesity, gut microbiota, endotoxemia, intestinal barrier

## Abstract

**Introduction:**

Thiamine (vitamin B1) in the gut is crucial for maintaining intestinal homeostasis and host health. Our previous study identified significantly lower levels of fecal thiamine in individuals with obesity; however, its potential and mechanisms for alleviating obesity induced by a high-fat and high-fructose diet (HFFD) remain unclear. Therefore, in the present study, the effects of high-dose thiamine supplementation on HFFD-induced obesity and gut microbiota dysbiosis were investigated.

**Methods:**

HFFD-fed mice were supplemented with high-dose thiamine for eight weeks. Biochemical analysis and histological analysis were conducted to assess phenotypic changes. Fecal 16S rRNA gene sequencing was performed to analyze alterations in the gut microbiota.

**Results:**

The results showed that high-dose thiamine supplementation for eight weeks could significantly alleviate symptoms of HFFD-induced obesity and improve HFFD-induced intestinal epithelial barrier dysfunction by enhancing the tight junction function. Furthermore, oral administration of high-dose thiamine also regulated HFFD-induced gut microbiota dysbiosis by reshaping its structure and composition of gut microbiota, such as increasing the relative abundance of *Actinobacteria* and *Bifidobacterium pseudolongum*, and reducing the relative abundance of *Proteobacteria* and *Ruminococcus gnavus*, accompanied by decreased level of gut-derived endotoxin. Finally, significant correlations were found between obesity-related phenotypes and gut microbiota through correlation analysis.

**Conclusion:**

Our findings suggest that the potential mechanism by which high-dose thiamine supplementation alleviated HFFD-induced obesity might involve reshaping gut microbiota and restoring the intestinal barrier, thereby ameliorating gut microbiota-related endotoxemia.

## 1 Introduction

Obesity is the excessive or abnormal accumulation of fat or adipose tissue in the body that impairs health via its association with the risk of development of diabetes mellitus, cardiovascular disease, hypertension, and hyperlipidemia (1). With its prevalence increasing dramatically over the past few decades, obesity has become a global health epidemic that continues to worsen (2–4). This complex disease has a multifactorial etiology (1). Generally, obesity is primarily driven by a myriad of genetic and environmental factors (1, 5).

Over the past decades, a growing body of evidence indicates that gut microbiota is an important environmental factor contributing to the onset and progression of obesity and related metabolic disorders (3, 6–8). Gut microbiota produce a diverse array of metabolites that influence energy metabolism, including short-chain fatty acids (SCFAs), bile acids, and different bioactive lipids (2). Notably, butyrate supplementation has been shown to alter gut microbiota composition and confer multiple metabolic benefits, including the prevention of high-fat diet-induced obesity (9). Targeting the microbiome has been emerging as a very attractive therapy for the treatment of obesity (7, 10).

B vitamins are essential micronutrients for both the host and gut microbiota, serving as biosynthetic precursors for universally essential cofactors used in numerous metabolic pathways (11, 12). For example, thiamine is required in amino acid and carbohydrate metabolism and is active in energy generation reactions (13); riboflavin acts as a precursor element of the flavin adenine dinucleotide and flavin mononucleotide, involved in electron balance during the production of aerobic energy (14); biotin acts as a carrier of carbon dioxide and plays a role in carboxylase enzymes involved in gluconeogenesis and fatty acid metabolism (15). B vitamins are considered to significantly contribute to intestinal homeostasis and host health (11, 12, 16, 17), and represent promising targets for reshaping microbial communities (18, 19). Studies have reported that supplementing with B vitamins or B vitamins-producing probiotics can modulate the gut microbiota, thereby affecting host health (16, 18, 20). For example, in the microbiota, the homeostasis of biotin metabolism and recycling is key for proper bacterial growth and function (21). Supplementing high-fat diet-fed mice with fructo-oligosaccharides and biotin improves not only the microbiome diversity but also the potential of bacterial production of biotin and B vitamins while limiting weight gain and glycaemic deterioration (21).

Thiamine (vitamin B1) is essential for the growth of microorganisms, thereby influencing the composition of gut microbiota (20). Gut microbiota is capable of producing thiamine on its own, which is crucial to the microbial community in the distal gut due to the efficient absorption of routine dietary vitamins in the small intestine (11, 19). Notably, when administered in large amounts beyond the threshold of small intestinal absorption, a portion of vitamins may escape absorption and directly modulate microbiota in the distal gut (22, 23). It has been reported that sufficient dietary thiamine intake had an influence on the gut microbial community (24). More importantly, it has been reported that high-dose thiamine could counter dyslipidemia in streptozotocin-induced diabetic rats (25) and had the potential to prevent obesity and metabolic disorders in Otsuka Long-Evans Tokushima Fatty rats (26). Our previous study observed a significantly lower fecal thiamine in obesity compared with healthy individuals (27). In agreement with our findings, a study conducted by Gao et al. (28) revealed a remarkable downregulation of thiamine metabolism in individuals with obesity. Recent studies also suggested that thiamine might be a drug candidate for the prevention of atherosclerotic cardiovascular disease in high-risk patients (29) and a potential therapeutic candidate for patients with gestational diabetes mellitus (30).

Although previous studies have provided valuable insights into the effects of thiamine on metabolic disease and gut microbiota, little is known about thiamine as a therapeutic agent to ameliorate diet-driven obesity by acting on the gut microbiota. Therefore, in the present study, a mouse model of HFFD-induced obesity was applied to explore the anti-obesity effect of high-dose thiamine supplementation by disease symptoms, intestinal barrier homeostasis, and gut microbiota.

## 2 Materials and methods

### 2.1 Animals and experimental design

**Figure 1:**
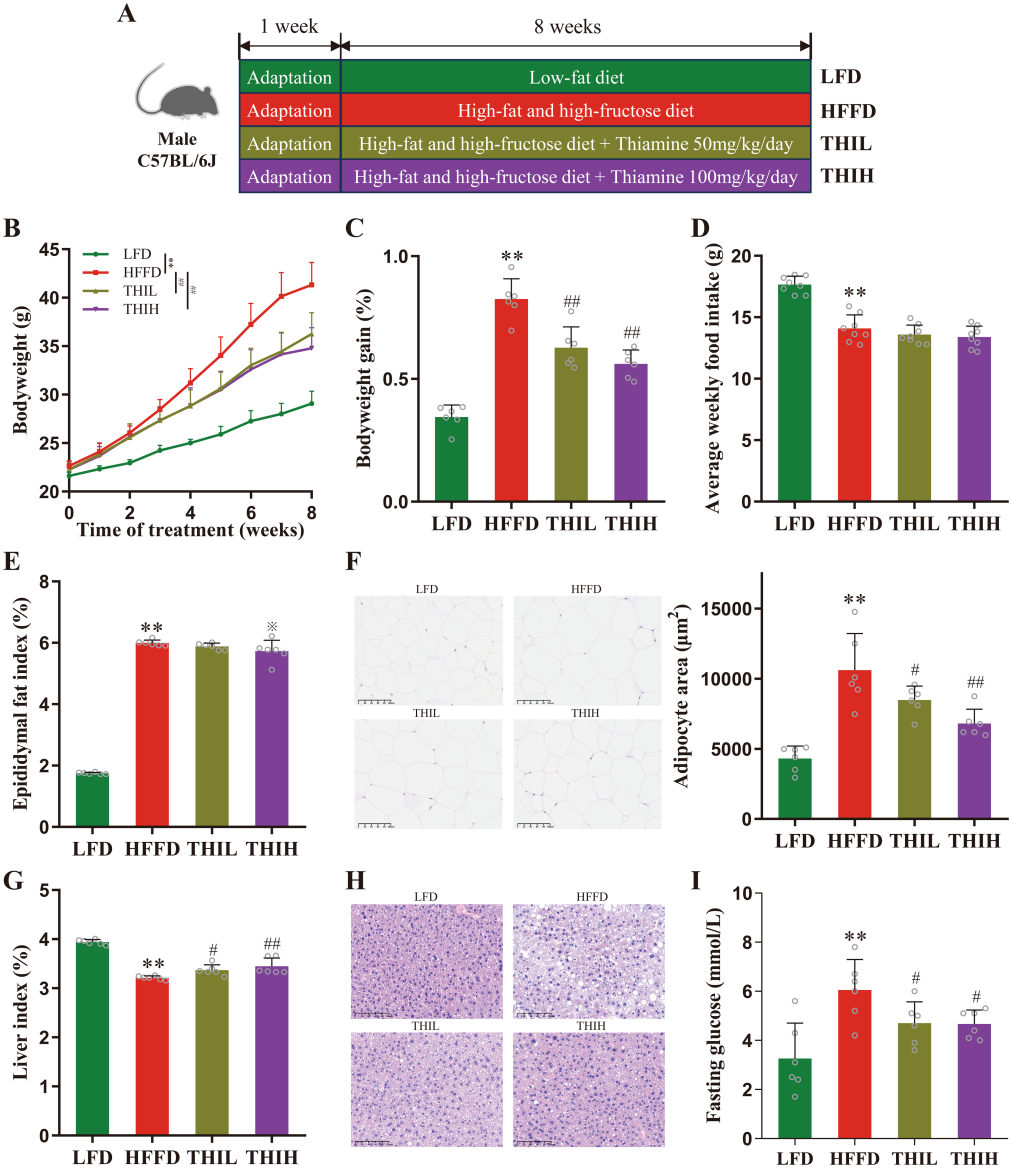
High-dose thiamine supplementation ameliorated HFFD-induced obesity. **(A)** Schematic diagram of the experimental design for the HFFD-induced obesity model and thiamine intervention. **(B)** Body weight of the LFD-fed mice and HFFD-fed mice treated daily with or without thiamine for 8 weeks. **(C)** Body weight changes of mice. **(D)** Average weekly food intake per mouse. **(E)** Epididymal fat index. **(F)** Representative pictures of hematoxylin and eosin (H&E)-stained white adipose tissue (scale bar, 100 μm) and adipocyte area. **(G)** Liver index. **(H)** Representative pictures of H&E-stained liver (scale bar, 100 μm). **(I)** Fasting serum glucose. Data were expressed as mean ± SD (*n* = 6). ^*^p < 0.05 and ^**^p < 0.01 vs. LFD group. ^※^*p* < 0.1, ^#^*p* < 0.05, and ^##^*p* < 0.01 vs. HFFD group.

Twenty-four male C57BL/6J mice, aged 5 weeks, were obtained from Gempharmatech Co., Ltd. (Nanjing, Jiangsu, China) and housed in a controlled, specific pathogen-free environment (21°C ± 2°C, 12-h dark–light cycle) with ad libitum access to food and water. After 1 week of acclimation on a normal diet, as shown in Figure 1A, 24 mice were randomly divided into four groups (*n* = 6 per group), including the low-fat diet (LFD) group fed with a low-fat diet (D12450J, Research Diets, Inc., United States), the high-fat high-fructose (HFFD) group fed with a high-fat diet (D12492, Research Diets, Inc., United States) plus 10% fructose water, and the low-dose thiamine group (THIL, 50 mg/kg/day, gavage) fed with HFD plus 10% fructose water, and high-dose thiamine group (THIH, 100 mg/kg/day, gavage) fed with HFD plus 10% fructose water. An oral supplement of 50 mg/kg or 100 mg/kg, corresponding to approximately 250 or 500 times the recommended dietary allowances for humans (31), respectively. The LFD group and the HFFD group administered the same volume of vehicle by intragastric gavage. The body weight of each mouse was measured using a calibrated digital scale at the beginning of the feeding period and weekly thereafter until euthanasia. Additionally, overall food intake for each group was monitored weekly throughout the study. The *in vivo* intervention lasted for 8 weeks (Figure 1A). At the end of the experiment, the mice, aged 14 weeks, were fasted for 12 h and then anesthetized in chambers saturated with isoflurane. The whole blood was collected through retroorbital sampling, after which the mice were sacrificed by cervical dislocation. Tissues, including adipose tissue, colon, and liver, were collected, weighed, and stored at −80°C for further analysis. The liver index was calculated as the ratio of liver weight to the body weight; while the epididymal fat index was calculated as the ratio of epididymal fat weight to the body weight. Animal experiments were conducted in accordance with the Guidelines for Animal Experimentation of China Pharmaceutical University (Nanjing, China), and the protocols were approved by the Animal Ethics Committee of this institution (No. 202407022).

### 2.2 Biochemical analysis

Serum samples were obtained after the blood samples were collected and centrifuged at 3,500 RPM for 15 min. Fasting serum glucose, triglycerides (TG), total cholesterol (TC), low-density lipoprotein cholesterol (LDL-C), high-density lipoprotein cholesterol (HDL-C) was measured by biochemistry automatic analyzer (Hitachi 7100, Japan). Serum endotoxin was assessed by commercial colorimetric kits (Xiamen Bioendo Technology, Xiamen, China) according to the manufacturer’s instructions (32).

### 2.3 Measurement of fecal thiamine

Fecal thiamine was measured by high-performance liquid chromatography-electrospray ionization-tandem mass spectrometry (HPLC-ESI-MS/MS) as we previously established (27).

### 2.4 Histological analysis

Livers, colon, and epididymal adipose tissues taken from the mice were was fixed with 4% paraformaldehyde overnight and embedded in paraffin wax. Then, the sections were sliced and stained with hematoxylin and eosin (H&E). Frozen liver sections were stained with Oil Red O and counterstained with hematoxylin to visualize the lipid droplets. The images of sections at 300 dpi resolution were captured using Leica Thunder Imaging System (Leica Microsystems). The area of adipocyte in epididymal fat tissue and the area of lipid droplets in liver tissue were determined by Image J software.

### 2.5 Immunofluorescence staining

The colon sections were incubated with 10% goat serum in phosphate-buffered saline (PBS) for 30 min. Subsequently, the primary antibodies, anti-zonula occludens-1 (anti-ZO-1, Servicebio, GB151981) diluted 1:2,000 and anti-occludin (Servicebio, GB111401) also diluted 1:2,000, were applied to the sections in a blocking solution and incubated overnight at 4°C. Following this, the sections were washed three times with PBS and treated with the specified fluorescein-labeled secondary antibody (Servicebio, GB23303) for 50 min. Cell nuclei were stained with 4,6-diamidino-2-phenylindole (DAPI) for 10 min and then washed three times with PBS. After sealing the slides with an anti-fluorescence quenching agent, images of the sections at 300 dpi resolution were captured using the Leica Thunder Imaging System (Leica Microsystems). The mean fluorescence intensity was determined by Image J software.

### 2.6 16S rRNA gene sequencing for gut microbiota analysis

Genomic DNA of fecal samples was extracted using MagBeads Fast DNA Kit for Soil (MP Biomedicals, CA, United States) and quantified using Nanodrop One spectrophotometer (Thermo Scientific). DNA was used to generate amplicons using a TruSeq Nano DNA LT Library Prep Kit. V3 and V4 hypervariable regions of prokaryotic 16S rDNA were selected for generating amplicons and following taxonomy analysis (32). DNA libraries were validated by Agilent High Sensitivity DNA Kit (Agilent Technologies, Palo Alto, CA, United States) and quantified by Quant-iT PicoGreen dsDNA Assay Kit. DNA libraries were multiplexed and loaded on an Illumina NovaSeq instrument according to the manufacturer’s instructions (Illumina, San Diego, CA, United States). Sequencing was performed using paired-end configuration; image analysis and base-calling were conducted by the Quantitative Insights Into Microbial Ecology 2 (QIIME2) Software. Kyoto Encyclopedia of Genes and Genomes (KEGG) analysis was performed using Phylogenetic Investigation of Communities by Reconstruction of Unobserved States (PICRUSt2) software. 16S rRNA gene sequencing data can be accessed on the Sequence Read Archive (SRA) database, accession number: PRJNA1188793.

### 2.7 Statistical analysis

Statistical analysis was performed using GraphPad Prism software (version 8). The results of biological assay are presented as mean ± standard deviation (SD). The differences between two groups were analyzed by Student’s *t*-test. Datasets that involved more than two groups were assessed by one way analysis of variance (ANOVA) followed by Tukey’s multiple comparison’s test. Permutational multivariate ANOVA (PERMANOVA) and principal-coordinate analysis (PCoA) were performed to analyze microbial β-diversity. The correlation between the microbiota and phenotypic characteristics was assessed using the multivariate linear regression test (MAASLIN). Additionally, correlations among the microbes were examined with Sparse Correlations for Compositional Data (SparCC). The statistical significance was set at *p* < 0.05.

## 3 Results

### 3.1 High-dose thiamine supplementation alleviated HFFD-induced obesity in mice

The effects of thiamine in preventing HFFD-induced obesity and the associated metabolic disorders were investigated by treating mice with high-dose thiamine for 8 weeks. As shown in Figures 1B,C, within 8 weeks of HFFD feeding, the body weight of mice in the HFFD group increased significantly compared with the LFD group, while oral administration of high-dose thiamine significantly inhibited HFFD-induced body weight gain after 4 weeks of thiamine administration. However, the weight loss effect was not observed to be dose-dependent between the THIL and THIH groups. No significant difference in food intake was observed among groups fed with HFFD, suggesting that the effects of thiamine were not due to reduced food consumption (Figure 1D and Supplementary Table S1). The epididymal fat index in HFFD-fed mice increased significantly than those in LFD-fed mice, and a trend toward a decreased epididymal fat index was observed in the THIH group, but not in the THIL group (Figure 1E and Supplementary Table S1). The histological analysis corroborated that thiamine intervention decreased epididymal fat deposits and adipocyte area (Figure 1F and Supplementary Table S1). In addition, thiamine intervention increased the liver index and alleviated the hepatic steatosis in liver tissues (Figures 1G,H and Supplementary Table S1). Furthermore, we found that the HFFD-induced hyperglycemia was attenuated by thiamine intervention, evidenced by a reduction of serum fasting glucose levels in thiamine-treat groups (Figure 1I and Supplementary Table S1).

### 3.2 High-dose thiamine supplementation alleviated HFFD-induced metabolic disorders in mice

**Figure 2:**
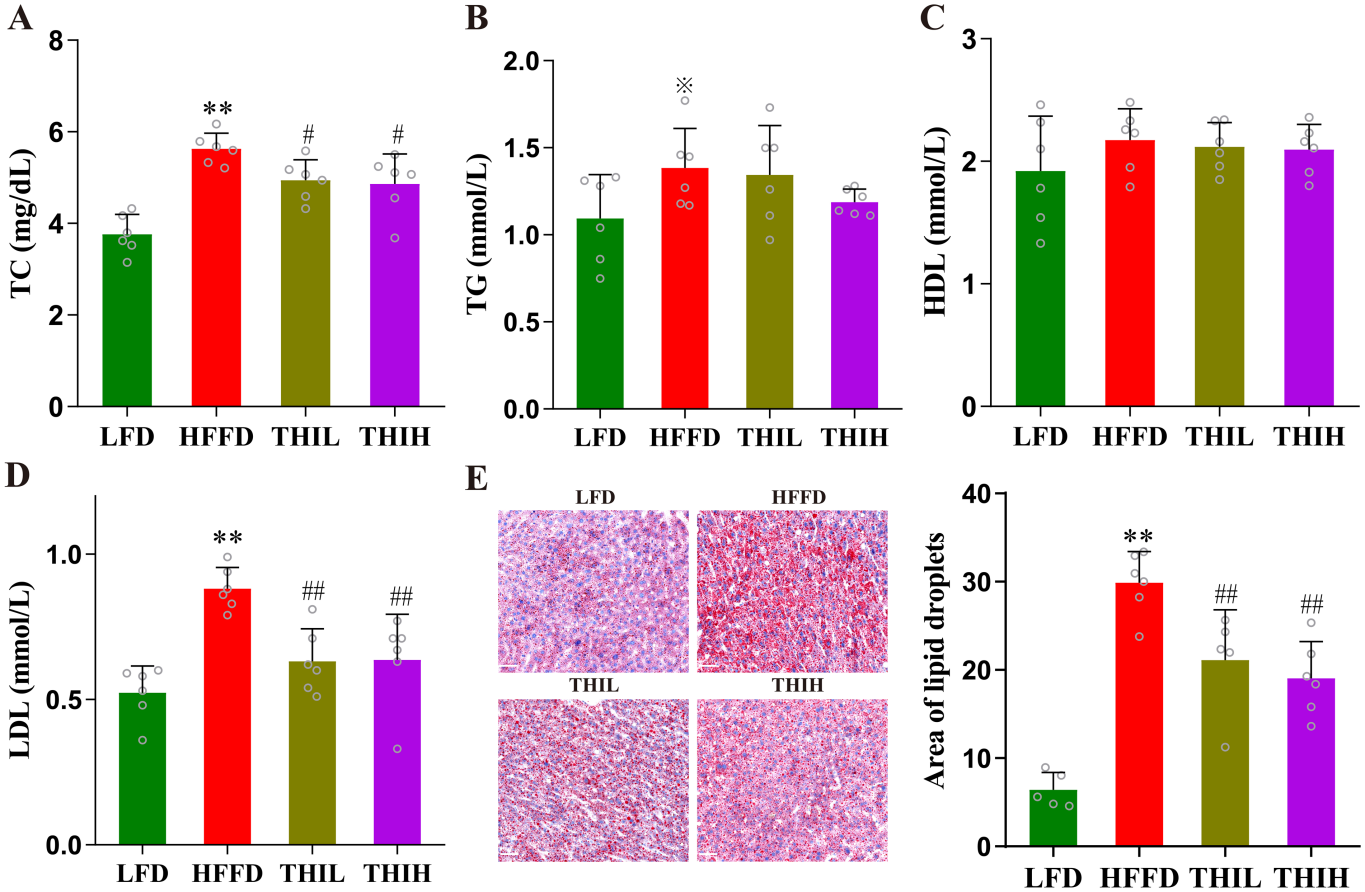
High-dose thiamine supplementation improved HFFD-induced metabolic disorders. **(A)** Serum TC. **(B)** Serum TG. **(C)** Serum HDL-C. **(D)** Serum LDL-C. **(E)** Liver lipid content was assessed using Oil Red O staining (scale bar, 50 μm) and area of lipid droplets. Data were expressed as mean ± SD (*n* = 5–6). ^*^p < 0.05 and ^**^p < 0.01 vs. LFD group. ^※^*p* < 0.1, ^#^*p* < 0.05, and ^##^*p* < 0.01 vs. HFFD group.

Compared to LFD feeding, HFFD feeding significantly altered lipid metabolism in mice, accompanied by adipose deposition, lipid accumulation in liver. However, these shifts were observed to be reversed by thiamine intervention. As shown in Figures 2A–D and Supplementary Table S1, the serum levels of TC and LDL-C were significantly reduced in the thiamine-treat groups compared with HFFD group. While, no significant changes in the serum levels of serum TG and serum HDL-C were observed in the thiamine-treated groups. Oil Red O staining revealed that lipid accumulation within the liver was reduced in thiamine-treat groups (Figure 2E and Supplementary Table S1).

### 3.3 High-dose thiamine supplementation alleviated HFFD-induced intestinal barrier dysfunction in mice

**Figure 3:**
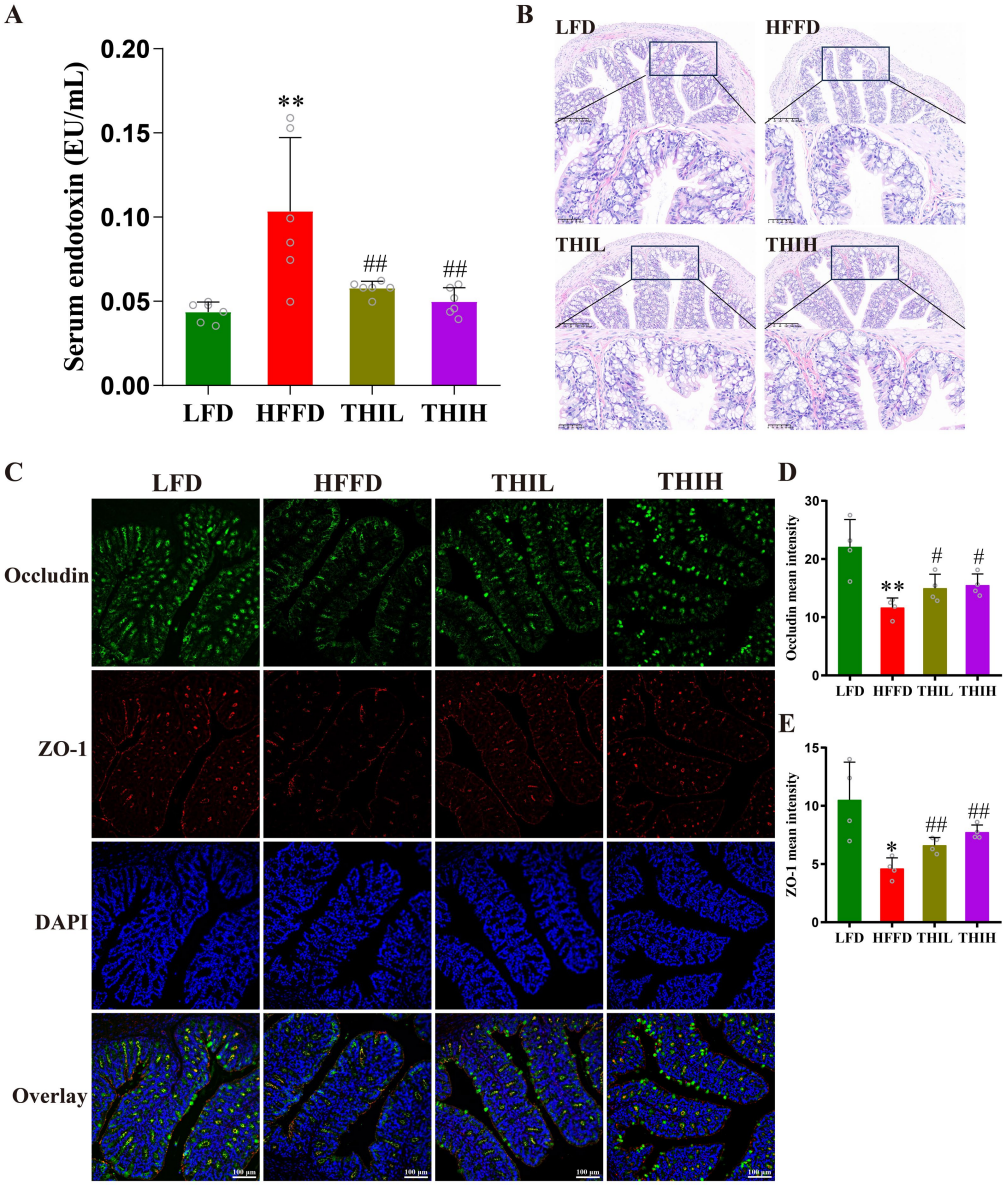
High-dose thiamine supplementation protected against the intestinal barrier damage caused by HFFD feeding. **(A)** Serum endotoxin (lipopolysaccharides, LPS) levels. **(B)** Representative images of H&E-stained colon tissue sections (scale bar, 50 μm and 200 μm). **(C)** Representative pictures of immunofluorescence staining of ZO-1 and occludin in the colon (scale bar, 100 μm). **(D)** Mean fluorescence intensity of occludin. **(E)** Mean fluorescence intensity of ZO-1. Data were expressed as mean ± SD (*n* = 4–6). ^*^p < 0.05 and ^**^p < 0.01 vs. LFD group. ^#^*p* < 0.05 and ^##^*p* < 0.01 vs. HFFD group.

Endotoxemia is a pivotal contributor to the onset and development of obesity. as shown in Figure 3A and Supplementary Table S1, we observed a higher serum endotoxin in HFFD group, whereas this increase was significantly reversed by thiamine intervention. The impairment of intestinal barrier is one of the important causes of endotoxemia; to further explore the effect of thiamine intervention on the histological damage in HFFD-induced obese mice, H&E staining was performed for histological examination of colon sections. The colon sections of healthy mice in LFD group exhibited a natural structure with a balanced distribution of goblet cells. However, compared to LFD group, HFFD feeding resulted in the destruction of crypt structures in the mucosa, edema in the submucosa, a reduction in goblet cells, and infiltration of inflammatory cells (Figure 3B). Notably, high-dose thiamine supplementation significantly alleviated these colonic structural damage and infiltration degree of inflammatory cells in HFFD-fed mice (Figure 3B).

Studies have shown that the integrity of the intestinal epithelial barrier is compromised in obesity, which is characterized by dysfunction of tight junctions. Tight junction proteins (e.g., ZO-1 and occludin) of epithelial cells are vital for the intestinal barrier integrity. To further explore the effect of thiamine intervention on intestinal barrier integrity, immunofluorescence staining analyses were performed to analyze the levels of intestinal tight junction proteins. As shown in Figures 3C–E, the levels of ZO-1 and occludin were significantly reduced in the HFFD-treated group compared to LFD group. Conversely, high-dose thiamine supplementation mitigated the intestinal barrier damage induced by HFFD feeding. These results suggest that thiamine intervention may positively regulate intestinal barrier permeability by preventing the decrease of tight junction protein levels in HFFD-induced obese mice.

### 3.4 Effects of thiamine intervention on the composition and function of gut microbiota in HFFD-induced obese mice

**Figure 4:**
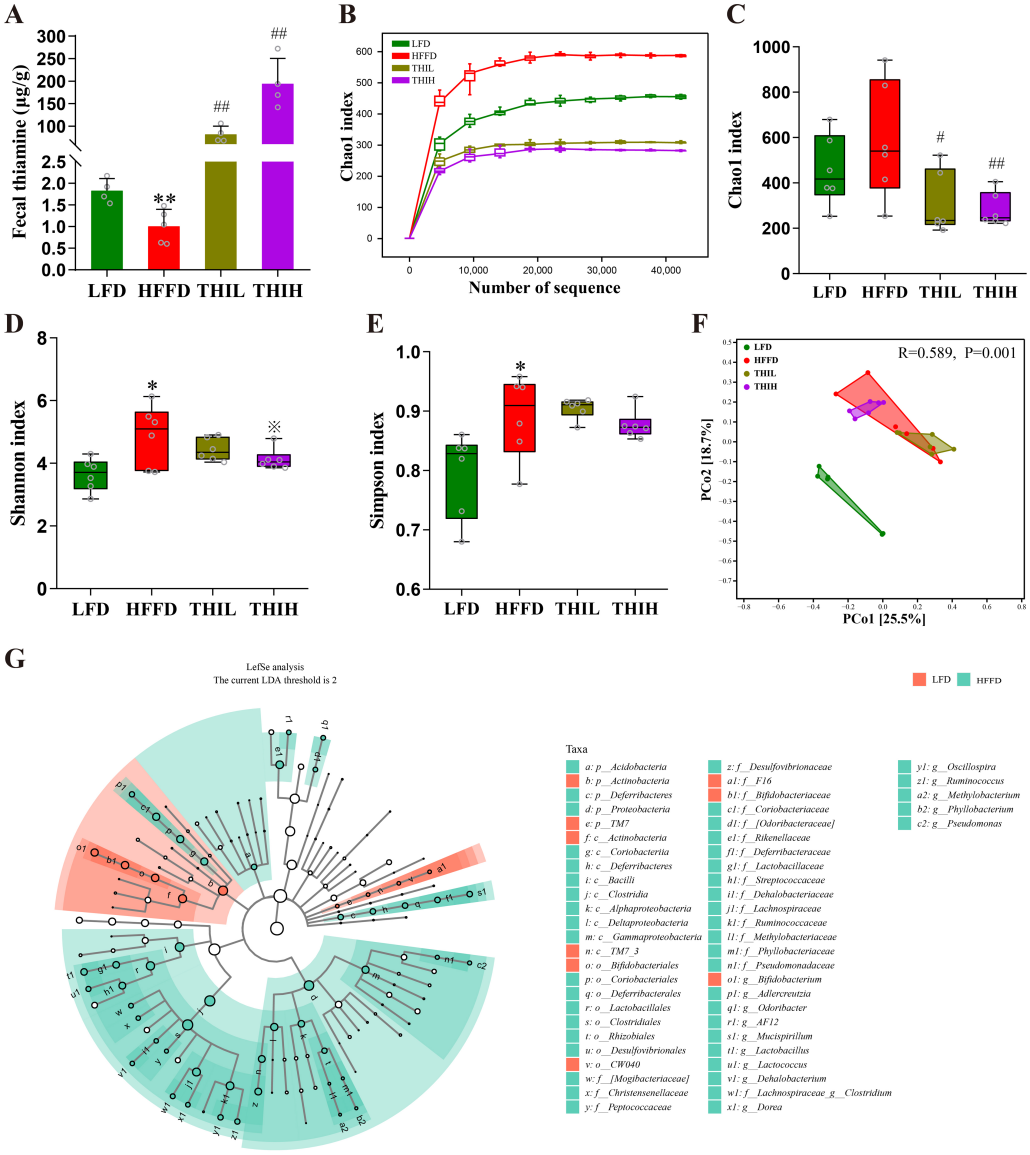
High-dose thiamine supplementation alleviated HFFD-induced gut dysbiosis. **(A)** Fecal thiamine levels. **(B)** Rarefaction curves based on Chao1 index. **(C)** Chao1 index. **(D)** Shannon index. **(E)** Simpson index. **(F)** Principal coordinate analysis (PCoA) of gut microbiota. **(G)** Cladograms generated by LEfSe indicating differences in the bacterial taxa between LFD group and HFFD group. ^*^p < 0.05 and ^**^p < 0.01 vs. LFD group. ^※^*p* < 0.1, ^#^*p* < 0.05, and ^##^*p* < 0.01 vs. HFFD group.

Impairment of intestinal barrier and endotoxemia is closely linked to gut microbiota disruption or dysbiosis. Notably, we observed that fecal thiamine levels in the HFFD group were significantly lower than those in the LFD group. In contrast, oral administration of high-dose thiamine resulted in a significant increase in fecal thiamine levels (Figure 4A). We next analyzed the composition and structure of fecal microbiota by 16S rRNA gene sequencing method to study the effect of thiamine on the gut microbiota of obese mice. As shown in Figure 4B, Chao1 index gradually reached a saturation plateau as the number of sequences increased, indicating that the amount of sequencing data is sufficient to reflect the most of the microbial information in the samples. Alpha diversity indices reflect the intrasample diversity of gut microbiota. As shown in Figures 4C–E and Supplementary Table S1, the gut microbiota structure of HFFD-feeding mice was characterized by increased Shannon index and Simpson index compared to the LFD group. Notably, a trend toward a decreased Shannon index was observed in the THIH group, but not in the THIL group. Furthermore, the Chao1 index was altered by thiamine intervention when compared to the HFFD group. Beta diversity reflects differences in species diversity among samples. The results of PCoA indicated a distinct separation gut microbiota community among the four groups (Figure 4F).

**Figure 5:**
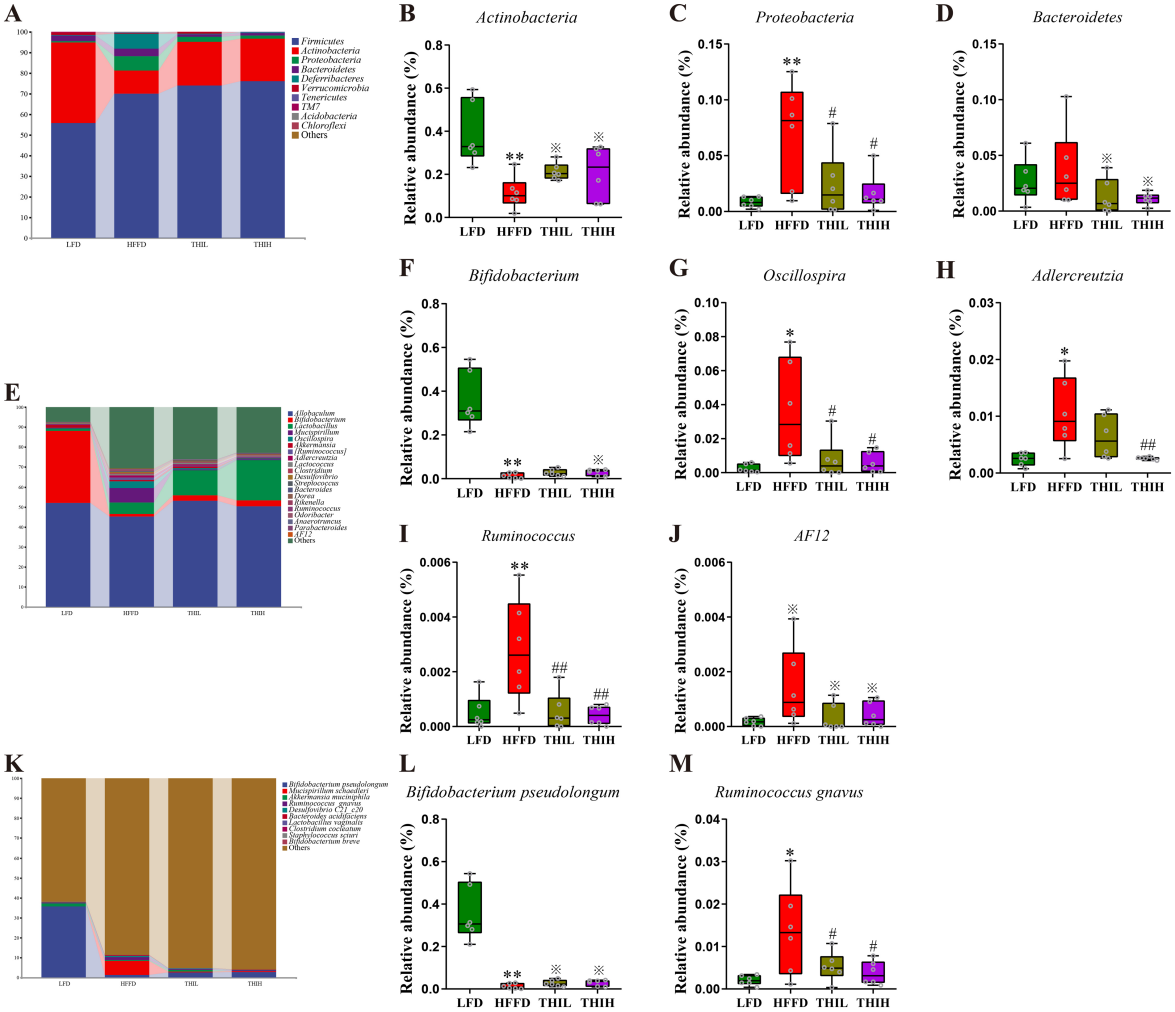
Effects of high-dose thiamine supplementation on gut microbiota composition and the relative abundance of specific microorganism in HFFD-induced obese mice. **(A)** Relative abundance of gut microbiota at the phylum level. **(B)** Relative abundance of *Actinobacteria*. **(C)** Relative abundance of *Proteobacteria*. **(D)** Relative abundance of *Bacteroidetes*. **(E)** Relative abundance of gut microbiota at the genus level. **(F)** Relative abundance of *Bifidobacterium*. **(G)** Relative abundance of *Oscillospira*. **(H)** Relative abundance of *Adlercreutzia*. **(I)** Relative abundance of *Ruminococcus*. **(J)** Relative abundance of *AF12*. **(K)** Relative abundance of gut microbiota at the species level. **(L)** Relative abundance of *Bifidobacterium pseudolongum*. **(M)** Relative abundance of *Ruminococcus gnavus*. ^*^p < 0.05 and ^**^p < 0.01 vs. LFD group. ^※^*p* < 0.1, ^#^*p* < 0.05, and ^##^*p* < 0.01 vs. HFFD group.

To identify the key gut microbes that most likely explain differences between HFFD group and LFD group, Linear Discriminant Analysis Effect Size (LEfSe) analysis was performed. Multiple taxonomic differences between two groups were identified (LDA score >2) (Figure 4G), indicating HFFD feeding had a dramatic influence on composition on the gut microbiota. We next examined the abundance of the aforementioned bacteria in the gut microbiota of each group at the phylum, genus, and species levels to further investigate the impact of thiamine intervention on the alterations of microbial composition induced by HFFD feeding. At the phylum level, gut microbiota in the four groups was mainly composed of *Firmicutes*, *Actinobacteria*, *Proteobacteria*, *Bacteroidetes*, *Deferribacteres*, *Verrucomicrobia*, *Tenericutes*, *TM7*, *Acidobacteria* and *Chloroflexi* (Figure 5A). Among them, we observed that the relative abundance of *Proteobacteria* was significantly boosted in the HFFD group, whereas thiamine intervention prominently reversed the relative abundance of that caused by HFFD feeding (Figure 5C and Supplementary Table S1). Thiamine intervention also exhibited a trend toward a decreased *Bacteroidetes* (Figure 5D and Supplementary Table S1). On the contrary, the relative abundance of *Actinobacteria* was significantly depleted in the HFFD group, whereas thiamine intervention prominently increased the relative abundance of that caused by HFFD feeding (Figure 5B and Supplementary Table S1). At the genus level (Figures 5E–J and Supplementary Table S1), we observed that the relative abundance of *Oscillospira*, *Ruminococcus* and *AF12* were significantly boosted in the HFFD group, whereas thiamine intervention prominently reversed the relative abundance of them caused by HFFD feeding. Notably, *Adlercreutzia* enriched in HFFD group only exhibited a significant reduction in THIH group, but not in the THIL group. On the contrary, the relative abundance of *Bifidobacterium* was significantly depleted in the HFFD group, while thiamine intervention exhibited a tendency to restore the relative abundance affected by HFFD feeding. At the species level (Figures 5K–M and Supplementary Table S1), we observed that the relative abundance of *Ruminococcus gnavus* was significantly boosted, whereas *Bifidobacterium pseudolongum* was significantly depleted in the HFFD group. Notably, thiamine intervention significantly reversed these changes.

We also investigated the potential alterations in functional pathways. As shown in Supplementary Figure S1, the PICRUSt analysis revealed several alterations in KEGG pathways between the LFD and HFFD groups. Notably, “fatty acid biosynthesis” and “beta-alanine metabolism” were enriched, while “protein digestion and absorption” and “other glycan degradation” were diminished in the HFFD group compared to the LFD group.

### 3.5 Correlation analysis of experimental parameters in HFFD-induced obesity mice

**Figure 6:**
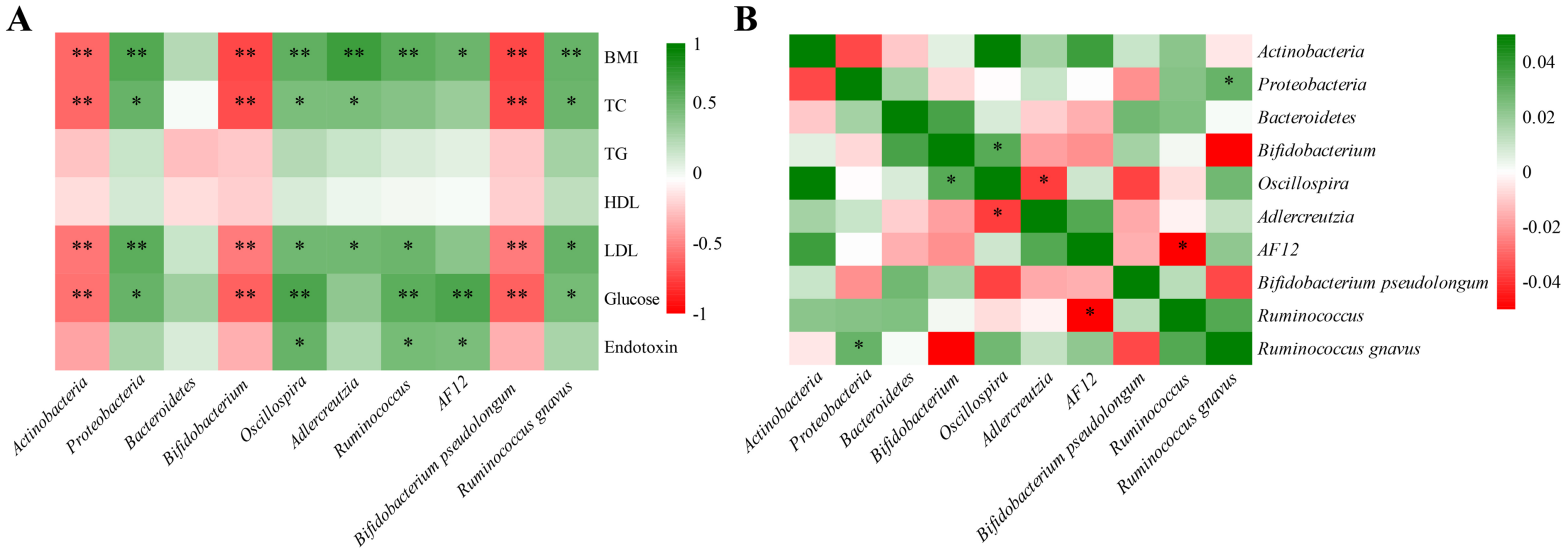
Correlation heat map of experimental parameters in HFFD-induced obesity mice. **(A)** Correlation heat map between the microbiota and phenotypic characteristics using MAASLIN. **(B)** Correlation heat map among the microbes using SparCC. ^*^Represents statistical significance, ^*^p < 0.05 and ^**^p < 0.01.

Correlation analyses were conducted to identify the relationships between gut microbiota and obesity-related phenotypes, as well as among the gut microbes. As shown in Figure 6A and Supplementary Table S2, the abundance of *Actinobacteria*, *Bifidobacterium*, and *Bifidobacterium pseudolongum* were negatively correlated with BMI, TC, LDL-C, and fasting serum glucose. In contrast, *Proteobacteria*, *Oscillospira*, and *Ruminococcus gnavus* exhibited positive correlations with BMI, TC, LDL-C, and fasting serum glucose. Additionally, as shown in Figure 6B and Supplementary Table S3, the abundance of *Proteobacteria* was positively correlated with *Ruminococcus gnavus*. Collectively, these findings suggest that high-dose thiamine mitigates HFFD-induced obesity by modulating specific gut microbiota.

## 4 Discussion

Obesity is one of the most prevalent chronic metabolic diseases with significant medical, social, and economic consequences, and it represents a major contributor to poor health in many countries (5, 33). Therefore, there is an urgent need for the development of effective and safe therapeutic agents. Gut microbiota dysbiosis has been extensively linked to obesity, rendering it a potential target for the treatment of obesity and associated comorbidities (7, 34, 35). B vitamins are essential micronutrients for both host and microorganisms (36), they are critical for maintaining the intestinal homeostasis (12, 16, 20). Dietary B vitamins are absorbed primarily in the proximal small intestine, and thus it is unlikely that they reach the distal gut, where densely populated microbial communities reside (19). Nevertheless, it has been reported that B vitamins can directly modulate gut microbiota when colon-targeted delivery or administered in large amounts (19). Our previous study observed significantly lower levels of fecal thiamine in individuals with obesity compared to healthy individuals (27). In the present study, we demonstrated that high-dose thiamine supplementation could significantly suppress the HFFD-induced body weight gain in the context of comparable food intake, and improve the disorder of glycolipid metabolism induced by HFFD feeding. Additionally, we illustrated that the potential mechanism of its anti-obesity effect involves reshaping gut microbiota and restoring the intestinal barrier, thereby alleviating gut microbiota-related endotoxemia.

Diet is one of the main factors shaping the gut microbiome (5), which plays a pivotal role in multiple phenotypes associated with obesity (10). It has been suggested that 60% of the gut microbial composition is determined by the host diet (37). Consistent with the previous reports, HFFD feeding leads to dramatic changes in gut microbial composition and structure, evidenced by the significant alterations in microbial α-diversity and β-diversity (38, 39). Our study demonstrated that high-dose thiamine supplementation in HFFD-fed mice restored the α- and β-diversity of the gut microbiota community observed in LFD-fed mice. Comparison of gut microbial composition between LFD and HFFD mice by 16S rDNA sequencing revealed multiple taxonomic differences from the phyla to genera. At the phylum level, HFFD feeding enriched the abundance of *Proteobacteria* while depleting *Actinobacteria*. *Proteobacteria* is the most consistently reported obesity-associated phylum (40). Studies have indicated that *Proteobacteria* often accompanied a high-at/high-sugar diet (41), and increased level of *Proteobacteria* is considered to be a potential indicator of dysbiosis and risk of disease (42). Correlation analysis revealed that the abundance of *Proteobacteria* was positively correlated with BMI, TC, LDL-C, and fasting serum glucose levels. Additionally, *Proteobacteria* is considered to be the main source of lipopolysaccharide (LPS) and is often associated with an increase in circulating LPS levels (34, 41). Notably, high-dose thiamine treatment restored this microbial shift in the HFFD group. At the genus level, HFFD feeding significantly enriched the abundance of *Ruminococcus*, *Adlercreutzia*, *Oscillospira*, and *AF*12, which could be restored by the thiamine intervention. Notably, *Ruminococcus* has been reported to increase with carbohydrate intake (43) and is considered to be an obesity-associated genus in studies from the West (40). Particularly, *Ruminococcus gnavus*, a prevalent mucolytic inflammatory gut microbe, is reproducibly associated with several features of metabolic syndrome in humans, including increased body fat percentage (44). In the present study, we found that *Ruminococcus gnavus* exhibited positive correlations with BMI, TC, LDL-C, and fasting serum glucose. A recent study revealed that *Ruminococcus gnavus* synergizes with HFD to promote glucose intolerance and hepatic steatosis potentially through rewiring of microbial tryptophan and phenylalanine metabolism (45). Our results revealed that high-dose thiamine supplementation could significantly reduce the abundance of *Ruminococcus gnavus* in HFFD-induced obese mice. Additionally, the HFFD feeding significantly down-regulated the relative abundances of *Bifidobacterium*, while thiamine intervention exhibited a trend toward an increased *Bifidobacterium*, particularly in the THIH group. *Bifidobacterium* is a widely recognized beneficial genus with health-promoting effects on the human host and is included in many probiotic preparations (46). Several studies have reported a correlation between a low abundance of *Bifidobacterium* spp. and obesity (47). In the present study, we found that *Bifidobacterium pseudolongum* was negatively correlated with BMI, TC, LDL-C, and fasting serum glucose. In addition, our study revealed that high-dose thiamine supplementation exhibited a trend toward an increased *Bifidobacterium pseudolongum*. Consistently, previous studies had also observed increased *Bifidobacterium pseudolongum* after prebiotic intervention in HFD-induced obese mice (48, 49). Furthermore, it has been reported that *Bifidobacterium pseudolongum* had the therapeutic potential for the treatment of obesity-related metabolic disorders (50). Together, these results indicate that high-dose thiamine supplementation could modulate the gut microbiome toward a healthier profile.

A diet high in fat has pro-inflammatory effects (34). Low-grade inflammation is recognized as one of the hallmarks of obesity and related metabolic disorders (34). Several studies have demonstrated that HFFD feeding could lead to increasing levels of gut microbiota-related LPS in the bloodstream (38). Chronically elevated levels of circulating gut-derived LPS or “metabolic endotoxemia” can result in sustained systemic inflammation, subsequently inducing metabolic disorders through the activation of Toll-like receptor 4 (TLR4) signaling (51, 52). In the present study, we likewise observed a significantly higher serum LPS in HFFD group compared with LFD group, consistent with the finding of enriched *proteobacteria* in HFFD-fed mice. Notably, our results demonstrated that high-dose thiamine supplementation could ameliorate HFFD-induced body weight gain and hepatic steatosis, decrease the levels of serum endotoxin, serum TC, serum LDL-C and fasting glucose in HFFD-induced obese mice.

In addition to the microbial overproduction of LPS in the gut, impaired integrity of the intestinal barrier can exacerbate LPS entering the bloodstream. Consistent with the previous reports (39), HFFD feeding significantly impairs the intestinal barrier, evidenced by the destruction of crypt structures in the mucosa, edema in the submucosa, a reduction in goblet cells, and infiltration of inflammatory cells in the HFFD group compared with the LFD group. Importantly, we observed that high-dose thiamine supplementation could promote intestinal barrier integrity and improve the impaired barrier function caused by epithelial damage and by dysregulation of tight junction proteins (ZO-1 and occludin). It has been reported that *Bifidobacterium* can interact with intestinal epithelial cell junctions to maintain the integrity of the intestinal barrier and inhibit LPS translocation; it can also protect the intestinal barrier from damage by maintaining the abundance of intestinal microbial species (53). A recent study found that *Bifidobacterium pseudolongum* was significantly correlated with enhanced intestinal barrier function induced by raspberry polysaccharides treatment (48). However, *Ruminococcus gnavus* has been considered to be closely linked to gut inflammation, as well as chronic inflammatory and metabolic diseases (54–56). Taken together, high-dose thiamine supplementation exhibited a positive effect on intestinal barrier protection by modulating the gut microbiota, and prevented gut microbiota-related endotoxin entry into the blood, which might explain its metabolic protective effect on one hand.

On the other hand, thiamine plays a vital role in a variety of metabolic reactions in all mammalian cells (57). Cellular deficiency/suboptimal levels of thiamine lead to impaired energy metabolism and increased oxidative stress; it also negatively impacts the normal physiology of mitochondria (57). Thiamine transporter-1 and -2 (THTR-1 and THTR-2) are well-characterized receptors that can uptake thiamine from both the small intestine and large intestine (58, 59). A recent study found that maternal high-fat diet during pregnancy could disrupt the balance of gut microbiota, leading to reduced levels of maternal gut microbiota-related thiamine, thereby resulting in impaired absorption of thiamine by down-regulating the protein expression level of thiamine transporter SLC19A3 (the gene encoding THTR-2). Intriguingly, these defects could be restored via thiamine supplementation (30). Another recent study demonstrated that exposure of gut epithelia to LPS could result in inhibition in thiamine uptake due to a decrease in the level of expression of its transporters (THTR-1 and -2) at the cell membrane that is likely mediated via a protein kinase A (PKA) signaling pathway (13). Additionally, diet-induced obesity is often accompanied by hypoxia in tissues and cells (60). It has been reported that hypoxia can cause a significant inhibition in thiamine uptake and a significant reduction in the expression of thiamine (SLC19A2 and SLC19A3) transporters and in the activity of their gene promoters (61). Taken together, it is reasonable to speculate that restoring thiamine absorption in colonocytes, thereby repairing the intestinal barrier, may be one mechanism by which high-dose thiamine supplementation ameliorates HFFD-induced obesity through the modification of gut microbiota. The detailed mechanism of action requires further investigation.

## 5 Conclusion

The present study revealed for the first time that high-dose thiamine supplementation effectively reduced body weight gain and improved glycolipid metabolism in HFFD-fed obese mice. Furthermore, high-dose thiamine supplementation reversed the gut microbiota dysbiosis induced by HFFD feeding, particularly by enriching beneficial *Bifidobacterium pseudolongum* and downregulating potentially pathogenic *Proteobacteria* and *Ruminococcus gnavus*. Additionally, high-dose thiamine supplementation could repair intestinal barrier damage, thereby protecting against gut microbiota-related endotoxemia in HFFD-fed obese mice. Collectively, these findings indicate that high-dose thiamine supplementation can mitigate obesity and related metabolic disorders by reshaping the gut microbiota in HFFD-fed obese mice. We believe that thiamine exhibits great potential as a novel therapeutic agent against HFFD-induced obesity in terms of its efficacy and safety. However, the detailed mechanisms by which high-dose thiamine supplementation repairs the intestinal barrier warrant further investigation in our future work.

## Data Availability

The datasets presented in this study can be found in online repositories. The names of the repository/repositories and accession number(s) can be found in the article/Supplementary material.
