# Supplementary figures and images for: High-dose thiamine supplementation ameliorates obesity induced by a high-fat and high-fructose diet in mice by reshaping gut microbiota
Source: Front Nutr. 2025 Feb 7;12:1532581. doi: 10.3389/fnut.2025.1532581 (PMC11842239; doi:10.3389/fnut.2025.1532581)

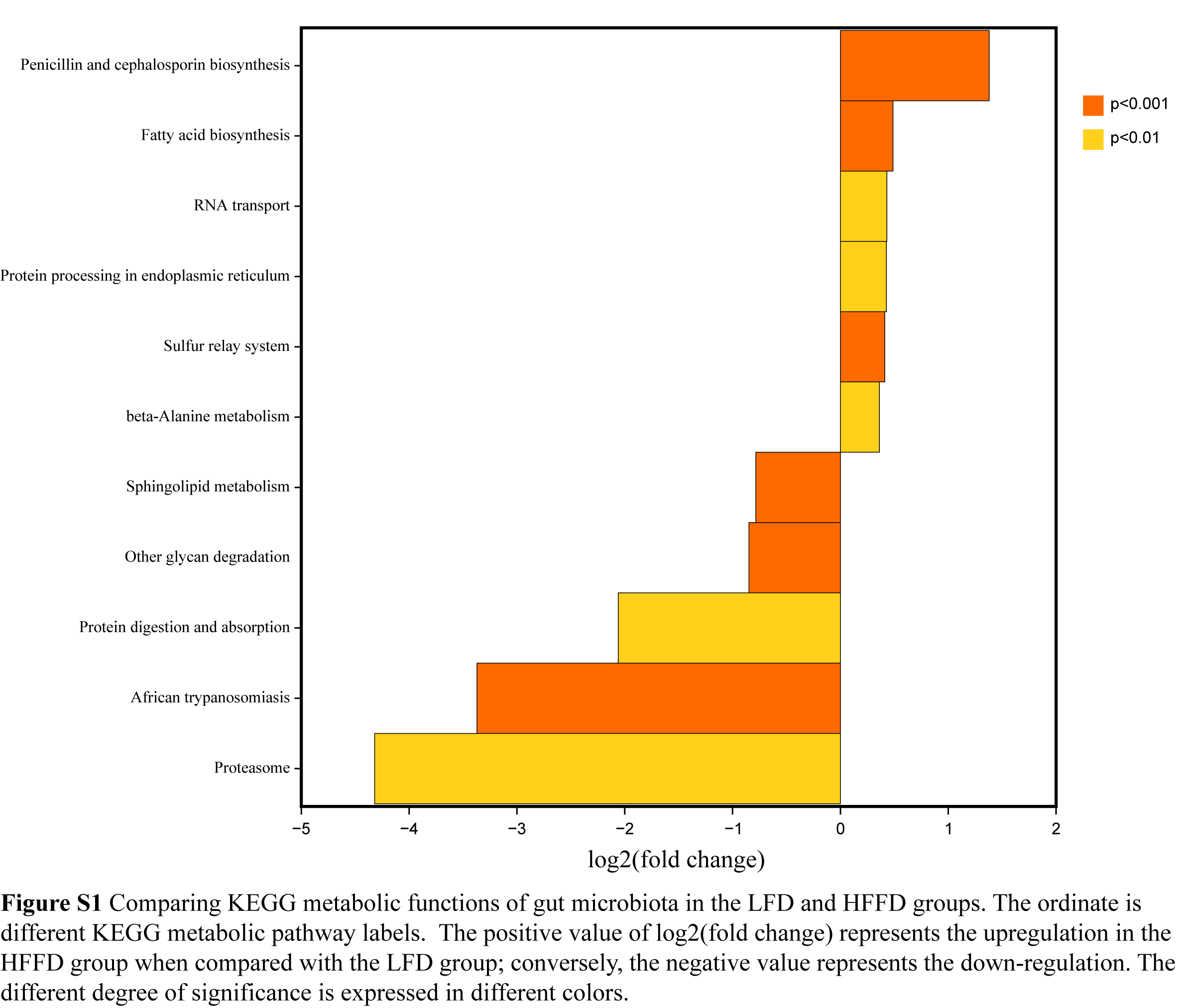

Supplement: Supplementary file 2 [file Image_1.tif]
